# The Functional Change and Deletion of *FLC* Homologs Contribute to the Evolution of Rapid Flowering in *Boechera stricta*

**DOI:** 10.3389/fpls.2018.01078

**Published:** 2018-07-31

**Authors:** Cheng-Ruei Lee, Jo-Wei Hsieh, M. E. Schranz, Thomas Mitchell-Olds

**Affiliations:** ^1^Institute of Ecology and Evolutionary Biology, National Taiwan University, Taipei, Taiwan; ^2^Institute of Plant Biology, National Taiwan University, Taipei, Taiwan; ^3^Genome and Systems Biology Degree Program, National Taiwan University, Taipei, Taiwan; ^4^Biosystematics Group, Wageningen University & Research, Wageningen, Netherlands; ^5^Department of Biology, Duke University, Durham, NC, United States

**Keywords:** *Boechera stricta*, *FLOWERING LOCUS C* (*FLC*), flowering time, gene duplication, repeated evolution

## Abstract

Differences in the timing of vegetative-to-reproductive phase transition have evolved independently and repeatedly in different plant species. Due to their specific biological functions and positions in pathways, some genes are important targets of repeated evolution – independent mutations on these genes caused the evolution of similar phenotypes in distantly related organisms. While many studies have investigated these genes, it remains unclear how gene duplications influence repeated phenotypic evolution. Here we characterized the genetic architecture underlying a novel rapid-flowering phenotype in *Boechera stricta* and investigated the candidate genes *BsFLC1* and *BsFLC2*. The expression patterns of *BsFLC1* suggested its function in flowering time suppression, and the deletion of *BsFLC1* is associated with rapid flowering and loss of vernalization requirement. In contrast, *BsFLC2* did not appear to be associated with flowering and had accumulated multiple amino acid substitutions in the relatively short evolutionary timeframe after gene duplication. These non-synonymous substitutions greatly changed the physicochemical properties of the original amino acids, concentrated non-randomly near a protein-interacting domain, and had greater substitution rate than synonymous changes. Here we suggested that, after recent gene duplication of the *FLC* gene, the evolution of rapid phenology was made possible by the change of *BsFLC2* expression pattern or protein sequences and the deletion of *BsFLC1*.

## Introduction

Disentangling the genetic architecture underlying evolutionary changes is a critical task for evolutionary geneticists, and the evolution of parallel phenotypic changes is particularly intriguing. For example, did independent evolution of the same phenotypes occur through *de novo* genetic changes in the same pathway, the same gene, or even the same nucleotide? Martin and Orgogozo (2013) reviewed many cases of genetic changes underlying phenotypic differences and found more than 100 cases describing “genetic hotspots” under repeated evolution.

Repeated evolution through the same gene could happen in two ways: independent gain or loss of function in separate lineages. The latter should be more frequent than the former because independent gain-of-function events often require the same mutation on the same functional site. Independent loss-of-function events, on the other hand, can happen in many ways, such as point mutation creating a premature stop codon, large insertions disrupting a gene, deletions removing the whole gene or important functional sites, or gene disruption by structural rearrangements. Recent gene duplication adds another level of complexity to this question. After gene duplication, duplicated homologs might undergo neo-functionalization or sub-functionalization, which will facilitate the gain of new functions. For loss-of-function events to happen, many possibilities exist depending on how the genes were duplicated. For example, if duplicated copies retain their original function, such functional redundancy makes further loss-of-function evolution difficult. On the other hand, one mutation in the original copy can cause loss of function if the duplicated copy does not retain the original function. This can happen if (1) only a portion of the gene was duplicated, creating a truncated and non-functional new copy, (2) the duplication event did not cover important regulatory regions flanking the gene, making the new copy cease to express in the same tissue or time, or (3) the new copy was intact and had the same spatiotemporal patterns of expression but later underwent amino acid substitutions changing the protein function. Note that while possibility (1) and (2) indicate a genetic change caused by the duplication event itself, possibility (3) focuses on changes after the duplication.

The onset of flowering marks the transition from vegetative to reproductive phases of plants and is under strong natural selection (Anderson et al., 2011; Munguía-Rosas et al., 2011). In some Brassicaceae, the requirement for vernalization before flowering is polymorphic, with vernalization requirement as the ancestral state. In *Arabidopsis thaliana*, this is mainly controlled by the natural variation of two floral suppressor genes, *FRIGIDA* (*FRI*) and *FLOWERING LOCUS C* (*FLC*), whose loss- or decrease-of-function mutations cause rapid flowering without vernalization requirement (Michaels and Amasino, 1999; Gazzani et al., 2003; Michaels et al., 2003; Lempe et al., 2005; Shindo et al., 2005; Werner et al., 2005). In this species, this phenotype has independently evolved from different mutations in the same genes (Johanson et al., 2000; Le Corre et al., 2002; Gazzani et al., 2003; Michaels et al., 2003; Lempe et al., 2005; Shindo et al., 2005; Werner et al., 2005; Méndez-Vigo et al., 2011; Li et al., 2014), constituting a good example of repeated evolution through the same gene.

Here we investigate the evolution of rapid lifecycle in *Boechera stricta* (Brassicaceae), a perennial wild relative of *Arabidopsis*. We ask whether parallel loss of vernalization responsiveness in a rapid-flowering phenotype of *B. stricta* involved mutations in one or both *FLC* homologs derived from a recent duplication event. We identified the candidate gene and its duplicated homolog, investigated their effects and expression patterns, and showed that the duplicated copy may have lost its flowering-related functions, allowing the evolution of rapid life cycle through deletion of the original *FLC* copy.

## Materials and Methods

### Plant Material and Flowering Time Estimation

*Boechera stricta* is a short-lived and predominantly self-fertilizing perennial native to the Rocky Mountains in North America. It has two subspecies (Lee and Mitchell-Olds, 2011; Lee et al., 2017) differing in many life history traits, with the EAST subspecies flowering faster than the WEST subspecies under most greenhouse and vernalization conditions (Lee and Mitchell-Olds, 2013). Previously, a major QTL, *nFT*, containing the candidate flowering time gene *FT*, was identified controlling phenological traits and local fitness in various environments (Anderson et al., 2011, 2012, 2014). Further investigations showed that the two parental alleles are identical in coding sequences but different in expression (Lee et al., 2014). In this study we investigated natural variation in vernalization requirement, which has not been previously studied in this species.

From a sample of ∼250 *B. stricta* accessions from the Northern Rocky Mountains (Lee and Mitchell-Olds, 2011), we identified one accession with very rapid flowering time from the EAST subspecies (family number 24B from the Moonrise Ridge population from Idaho, 44° 39′ N, 114° 32′ W, hereafter MR24). In the absence of vernalization in the greenhouse a typical *B. stricta* plant did not flower until 4–6 months, whereas the MR24 accession flowered within 2 months. We chose 14 accessions from the Moonrise Ridge population and performed complete randomized block experiments in the greenhouse to characterize flowering phenology with or without vernalization. To minimize maternal effects, we used seeds from accessions that were grown in the greenhouse for at least one generation. The greenhouse experiment consisted of 14 accessions, with seven from the faster and seven from the slower phenology groups. Each block contained 84 individuals with six plants from each accession, where three individuals were assigned to vernalization and three to the non-vernalization treatment. A total of seven blocks (588 plants) were used in this experiment. Like *A. thaliana, B. stricta* is a naturally self-fertilizing species, and sibling plants of the same accession were treated as replicated genetic clones throughout this study. Seeds were stratified in 4°C for 4 weeks, and seedlings were grown in “cone-tainer” racks (Stuewe & Sons Inc., Tangent, OR, United States) in the same environment (16-h days and 20°C ambient temperature) described in Lee and Mitchell-Olds (2013). When 2-month old, plants in the vernalization treatment were moved to 4°C, 10-h days for 6 weeks, and plants in the non-vernalization treatment remained in the greenhouse. The vernalization treatments for all following experiments were performed under the same condition (4°C, 10-h days) to simulate field environments during late winter and early spring. Since such treatment changed both temperature and day length, we recognize the caveat that we may not be able to separate effects from the two factors on flowering. We recorded flowering time (days to first flower) and leaf number when flowering. For plants with vernalization, the duration under vernalization was subtracted from the estimation of flowering time, thus recording the number of days under warm conditions. The data were analyzed with mixed-effect ANOVA using days to first flowering or leaf number when first flowering as the response variable. Treatment (vernalization/non-vernalization) was used as fixed effect. Block, accession, and accession by treatment interaction were treated as random effects in JMP 8 (SAS, Cary, NC, United States).

### Candidate Gene Profiling for Rapid Phenology

To identify candidate genes controlling the rapid phenology of MR24, we compared MR24 with a local accession (MR7A), whose flowering was significantly accelerated by vernalization. Using genomic DNA and cDNA from rosette leaves, we performed PCR and reverse transcription PCR (RT-PCR) on *FLC*, an important gene controlling the vernalization requirement in *Arabidopsis* (Michaels and Amasino, 1999; Sheldon et al., 2000; Shindo et al., 2005). All primer sequences are available in Supplementary Table S1. A previous study identified two *FLC* homologs in the *Boechera* genome (Schranz et al., 2007). This gene duplication is not due to polyploidy or tandem duplication, but rather by a transposition duplication (Lee et al., 2017). *BsFLC1* locates in the ancestral syntenic position (Scaffold13175 on chromosome 6) with *Arabidopsis FLC*, while *BsFLC2* was duplicated and transposed to Scaffold18351 on *B. stricta* chromosome 5. Based on this information, we designed copy-specific PCR primers for *B. stricta FLC* RT-PCR (*BsFLC1*: primer FLC-B and FLC1-C, *BsFLC2*: primer FLC-B and FLC2-C, Supplementary Table S1). To further investigate whether the lack of *BsFLC1* PCR amplification is due to a missing *BsFLC1* or mutations in primer binding sites, we used three forward primers conserved between *BsFLC1* and *BsFLC2* (FLC-B, FLC-F, FLC-G, Supplementary Table S1) and three *BsFLC1*-specific reverse primers (FLC1-C, FLC1-D, FLC1-E, Supplementary Table S1) to generate nine PCR amplicons for the genomic DNA of *BsFLC1*.

We further used Nanopore long-read sequencing technology to identify the extent of deletion. Genomic DNA of the MR24 accession was extracted using a modified CTAB protocol (Doyle and Doyle, 1987), and the library was made with the Nanopore Rapid Sequencing Kit (SQK-RAD004) and sequenced in one R9.4 flow cell. All library preparation and sequencing steps were performed according to standard Nanopore protocols. Resulting reads in fastq format were mapped to the *B. stricta* reference genome v1.2 (Lee et al., 2017) using GraphMap (Sović et al., 2016) and visualized with Integrative Genomics Viewer (Robinson et al., 2011). We designed primer pairs spanning the inferred chromosomal deletion and confirmed the deletion with PCR and Sanger sequencing.

### Test of *BsFLC1* Effects on Flowering Time

To test whether the lack of *BsFLC1* co-segregated with rapid phenology in progeny of accession MR24, we performed a controlled cross between MR24 (with the *BsFLC1* deletion) and MAH (with a functional *BsFLC1* and requiring vernalization). From a self-fertilized hybrid F_1_ we obtained 1,000 F_2_ individuals. These seeds, along with 39 replicated individuals from each parental accession, were stratified in 4°C for 4 weeks, and seedlings were randomized and grown in the greenhouse without vernalization.

The presence or absence of the *BsFLC1* PCR amplicon is a dominant marker in this cross (both MAH homozygotes and heterozygotes have visible *BsFLC1* amplicon on agarose gel). We identified one polymorphic microsatellite co-dominant marker (marker JGI13175-36 at around 513 kb in Scaffold13175, 73 kb away from *BsFLC1*) and genotyped this marker in 384 F_2_ individuals for the effect of *BsFLC1*.

### Comparing the Expression Patterns and Molecular Evolution of *BsFLC* Copies

To characterize the possibly differentiated functions between these two *BsFLC* copies, we first investigated the expression profiles of both *BsFLC* copies as well as *FT* and *SOC1*, two floral pathway integrator genes that are directly inhibited by *FLC* in *Arabidopsis* (Helliwell et al., 2006). Multiple individuals from two accessions (MAH with functional *BsFLC1* and MR24 with *BsFLC1* deletion) were used in the experiment. Seeds were stratified in 4°C for 4 weeks. Plants were randomized and grown in the greenhouse for 7 weeks and separated into two experimental groups, either with or without 6-week vernalization in 4°C under 10-h days. One of the target genes, *FT*, exhibits circadian rhythm in gene expression in *Arabidopsis*, and under 16-h days its maximum expression is in leaves in the end of daytime (Yanovsky and Kay, 2002; Cheng and Wang, 2005; Kim et al., 2008). In addition, *FLC* and *SOC1* also express in *Arabidopsis* leaves (Lee and Lee, 2010). We therefore used leaf tissues collected around 22:00 h (the sunset of 16-h days in the greenhouse) for expression profiling. Young leaves from four individuals of each accession were collected from six time points (Supplementary Figure S1: BV – before vernalization, when plants were 7 weeks old; NV1 – no vernalization group 1, when plants were 9 weeks old, and MR24 flowered but MAH did not; NV2 – no vernalization group 2, when plants were 18 weeks old, and MR24 had mature and dehiscing siliques but MAH had first flowers; AV1 – after vernalization group 1, 1 week after vernalization when MR24 flowered but MAH did not; AV2 – after vernalization group 2, 3.5 weeks after vernalization when MR24 had flowers and fruits but MAH had only flowers; AV3 – after vernalization group 3, 6 weeks after vernalization when both accessions had only fruits). We used Sigma Spectrum^TM^ Plant Total RNA Kit for RNA extraction and Thermo Scientific DyNAmo cDNA Synthesis Kit for cDNA synthesis, resulting in 47 separate samples (six time points with two accessions and four individual plants each, where one sample was lost during RNA extraction).

qPCR from cDNA was performed for five genes (*BsFLC1, BsFLC2, FT, SOC1, ACT2*), with *ACT2* as the reference gene. For each sample, gene expression was estimated with the ΔCt method:

$$\begin{array}{c} \mathrm{Relative}\;\mathrm{expression}\;\mathrm{to}\;ACT2\;=\;2^{\wedge }({\mathrm{Ct}}_{ACT2}\;-\;{\mathrm{Ct}}_{\mathrm{Gene}}), \end{array}$$

where Ct*_ACT2_* is the Ct value of *ACT2*, and Ct_Gene_ is the Ct value of target genes (*BsFLC1, BsFLC2, FT*, or *SOC1*). Using expression relative to *ACT2* as the response variable, statistical analyses (ANOVA) were conducted separately for each target gene, where genotype (MR24 or MAH), time point (BV, NV1, NV2, AV1, AV2, or AV3), and their interaction were treated as fixed effects in JMP 8 (SAS, Cary, NC, United States). To analyze the correlation of gene expression across the six time points, for each genotype separately, we further calculated the pairwise Spearman’s rank correlation coefficient ρ between the four target genes.

To characterize the evolutionary history of the two *BsFLC* copies, we cloned and sequenced their full-length coding sequences in *B. stricta* accessions MR24, MAH, and LTM (accession for the reference genome). The sequences were deposited in GenBank under the accession numbers MH166767–MH166771. RNA was extracted with “Sigma Spectrum^TM^ Plant Total RNA Kit” from rosette leaves, and cDNA was synthesized with “Life Technologies ThermoScript^TM^ RT-PCR System for First-Strand cDNA Synthesis.” cDNA was amplified with conserved primers for both *FLC* copies (FLC-H in 5′ UTR and FLC-I in 3′ UTR, Supplementary Table S1 and Supplementary Figure S2) and cloned in “Thermo Scientific CloneJET PCR Cloning Kit.” Full-length coding sequences of other Brassicaceae species were obtained from GenBank: *Arabidopsis arenosa FLC1* (DQ167446) *FLC2* (DQ167444), *Arabidopsis halleri* (AB465585), *Arabidopsis suecica* (DQ167447), *Brassica napus FLC1* (AY036888) *FLC2* (AY036889) *FLC3* (AY036890) *FLC4* (AY036891) *FLC5* (AY036892), *Brassica rapa* (EF460819), *Brassica oleracea* (AY273161), *Capsella rubella* (JQ993010), *Cardamine flexuosa* (KC618318), *Eutrema wasabi* (HM639741), *Sinapis alba* (EF542803), *Thellungiella halophila* (AY957537), and *Raphanus sativus* (JX050205).

*FLC* coding sequences of Brassicaceae were examined in MEGA 4 (Tamura et al., 2007). A phylogenetic tree was reconstructed in MrBayes v3.1.2 (Ronquist and Huelsenbeck, 2003) with generalized time reversible model allowing invariable sites and gamma distribution for evolutionary rate difference among sites. Two independent runs were performed with eight chains. Trees were sampled every 1,000 generations, and the first 500 trees were discarded, leaving 1,500 trees for the tree topology summary. We only kept the tree topology and branch support from MrBayes (Rannala et al., 2012), and branch lengths were estimated with maximum likelihood in PAUP^∗^ 4.0b10 (Swofford, 2003) with the same model described above.

Our results showed that the two *BsFLC* copies in *B. stricta* were generated by a gene duplication event after *Boechera* diverged from *Arabidopsis* and *Capsella*. We therefore used *Arabidopsis FLC* as the outgroup to analyze patterns of molecular evolution after *BsFLC* duplication. The ancestral sequence before gene duplication was reconstructed using PAML 4 (Yang, 2007), and synonymous and nonsynonymous substitutions were estimated with DnaSP v5 (Librado and Rozas, 2009). We further used PAML 4 to test three hypotheses: (1) Whether the *d_N_*/*d_S_* values are significantly different between *BsFLC1* and *BsFLC2* (using the branch model, where the *d_N_*/*d_S_* values for the two genes are the same in the null hypothesis but different in the alternative hypothesis); (2) Whether the *d_N_*/*d_S_* value for *BsFLC2* is significantly higher than one, showing signs of positive selection (using the branch model, where the *d_N_*/*d_S_* value for *BsFLC2* was restricted to one in the null hypothesis but unrestricted in the alternative hypothesis); and (3) Was there any codon site under strong positive selection on the *BsFLC2* branch (the branch-site model, Zhang et al., 2005).

We used custom R scripts (R Core Team, 2014) to investigate whether amino acid substitutions accumulated on *BsFLC2* after gene duplication occurred randomly on the protein or clustered in a specific functional domain. Among the total of 196 amino acids in this protein, we randomly draw nine “substitutions” (*BsFLC2* accumulated nine substitutions after duplication) and recorded the number of substitutions inside the K-box domain. This procedure was repeated 1,000 times, and the observed value was compared with the distribution of 1,000 K-box-specific substitutions under the assumption that substitutions occurred randomly along the protein.

## Results

### High Genetic Variation for Phenology Within a Population

**FIGURE 1:**
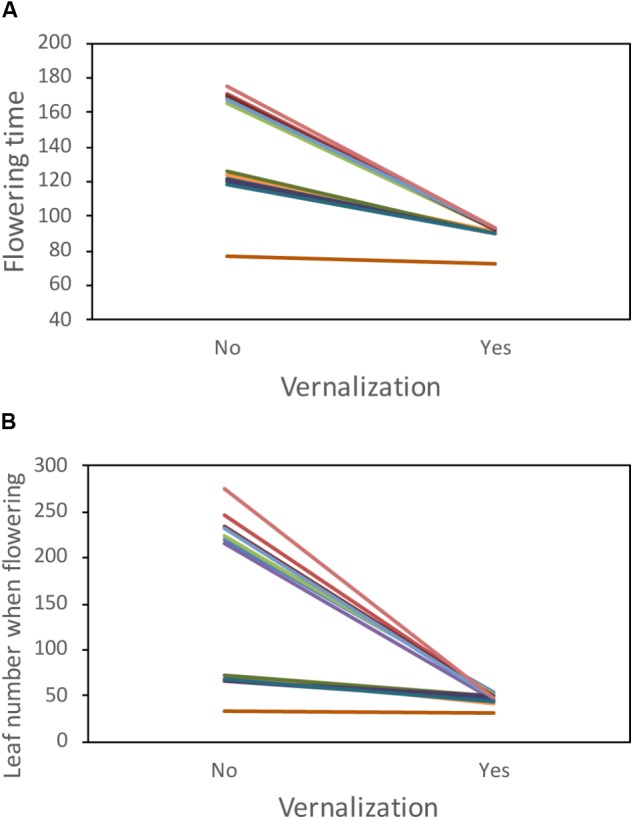
Phenology of accessions from the Moonrise Ridge population. **(A)** First flowering time in days after germination and **(B)** leaf number when first flowering. Each colored line represents one accession. One accession, MR24, has rapid flowering regardless of vernalization treatment.

Accessions from the Moonrise Ridge population showed highly significant differences in their phenology and response to vernalization treatment. Specifically, treatment and accession-by-treatment interaction had highly significant effects on both flowering time and leaf number at first flower (**Figure 1** and **Table 1**). Notably, while vernalization accelerated flowering in most accessions, the accession MR24 exhibited rapid flowering regardless of the vernalization treatment. This lack of vernalization requirement in MR24 is unusual in *B. stricta*, and we focused on the genetic mechanism underlying this trait.

**Table 1:** Analysis of variance of phenology in the Moonrise Ridge population.

	Flowering time		Leaf number when flowering	
Factor (effect)	Statistic^a^	*P*-value	Statistic	*P*-value
Vernalization (fixed)	*F*_1,13_ = 54.71	<0.001	*F*_1,13_ = 18.47	<0.001
Accession (random)	*x*^2^_1_ = 1.01	0.315	*x*^2^_1_ = 0.05	0.822
Accession ^∗^Vernalization (random)	*x*^2^_1_ = 980.27	<0.001	*x*^2^_1_ = 545.26	<0.001
Block (random)	*x*^2^_1_ = 0	1.000	*x*^2^_1_ = 6.60	0.010

*^*a*^Significance of random effects was determined by likelihood ratio test between models with or without these effects. Subscript numbers after F denote the numerator and denominator degrees of freedom, and the subscript number after x^*2*^ denotes degrees of freedom for likelihood ratio test.*

### Candidate Gene Profiling Odentified *BsFLC1* as Possible Causal Gene

**FIGURE 2:**
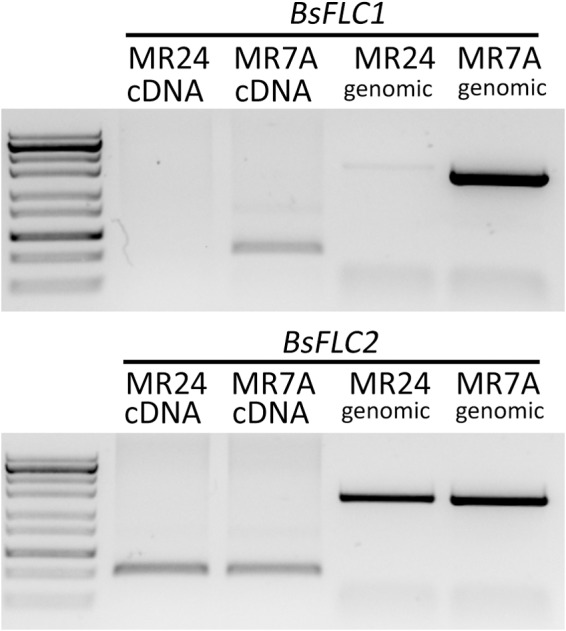
Candidate gene profiling of two *FLC* copies. Labels for each gene (*BsFLC1* or *BsFLC2*), accession (MR24 or MR7A), and PCR template (cDNA or genomic) are above each lane. RNA was extracted from leaves in the rosette stage grown in 16-h day greenhouse condition. Ladder band from top to bottom (kb): 10, 5, 3, 2, 1.2, 0.85, 0.5, 0.3, 0.1.

Since the flowering of MR24 was not affected by vernalization, we investigated homologs for the candidate genes *FLC* (the key gene underlying the vernalization requirement in *Arabidopsis*) in the rapid-flowering MR24 and slow-flowering MR7A accessions. Previous work identified two *FLC* copies in *Boechera* (Schranz et al., 2007), and these two copies showed distinct patterns between MR24 and MR7A (**Figure 2**): while *BsFLC2* is present and expressed in both accessions, the *BsFLC1* locus was only amplified in the slow-flowering MR7A but not the rapid-flowering MR24 accession. To test whether this lack of *BsFLC1* amplification in MR24 was due to deletion of *BsFLC1* or mutation in primer binding sites, three forward and three reverse primers were used to amplify *BsFLC1*, and products from all nine primer combinations were successfully amplified in MR7A but not in MR24. We therefore conclude that *BsFLC1* was lost in the rapid-flowering MR24 accession. *FLC* in *Arabidopsis* functions as a floral repressor and is suppressed by vernalization, and the lack of *BsFLC1* in MR24 is consistent with its rapid-flowering phenotype regardless of vernalization treatment.

We used the Nanopore long-read sequencing technology to confirm and investigate the extent of this deletion. We identified three reads supporting the chromosomal deletion. Further PCR and Sanger sequencing from primer pairs spanning the deletion breakpoint confirmed this finding (Supplementary Figure S3). This deletion covers *BsFLC1* and is about 19-kb long, ranging from 435.5 to 545.6 kb on Scaffold13175 of the reference genome. In addition, another upstream ∼9 kb deletion was identified by a long read (Supplementary Figure S3). This region might be an accession-specific insertion in the LTM reference genome, as we did not find this indel in the alignment between this long Nanopore read and the *Boechera retrofracta* (a sister species of *B. stricta*) assembly (Kliver et al., 2018).

### The Effect of *BsFLC1* on Flowering Time

**FIGURE 3:**
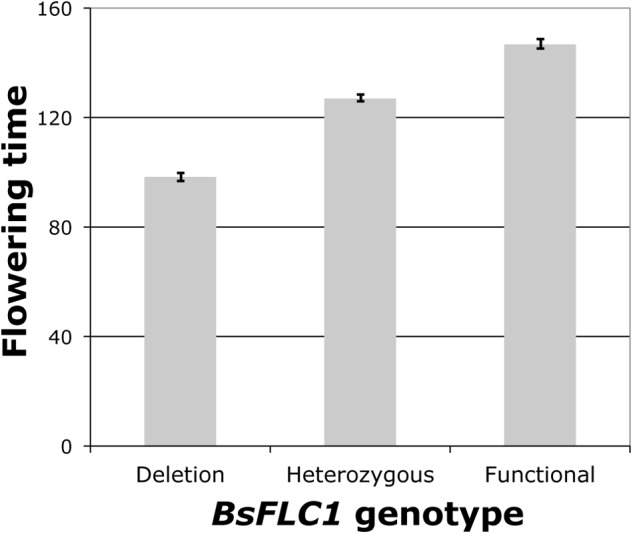
*BsFLC1* deletion significantly accelerates flowering (*P* = 10^-61^) in 384 F2 individuals. Bars represent the mean and standard error of the days to first flower in three genotypes of a microsatellite marker 73 kb from *BsFLC1*. All three bars are significantly different under the Tukey’s Honest Significant Difference test.

To estimate the effect of the *BsFLC1* deletion, flowering time was measured in 1,000 F_2_ plants (Supplementary Figure S4) from a cross between parents MR24 (with *BsFLC1* deletion) and MAH (with functional *BsFLC1*), and 384 plants were genotyped for a microsatellite marker (JGI13175-36) ∼73 kb from *BsFLC1* in the reference genome and ∼58 kb from the deletion in the MR24 genome. From the re-sequencing of 159 F_6_ recombinant inbred lines (Lee et al., 2017), no recombination event was observed between *BsFLC1* and this microsatellite marker. The microsatellite had highly significant association with flowering time (**Figure 3**, *P* = 10^-61^), accounting for about 70% of phenotypic variation in the F_2_ plants, and the number of functional *BsFLC1* alleles showed roughly additive effects on flowering time (**Figure 3**), consistent with other Brassicaceae species (Schranz et al., 2002). Our results are therefore consistent with the hypothesis that the *BsFLC1* deletion is associated with the rapid phenology in MR24 accession.

### Expression Patterns of *BsFLC1, BsFLC2, FT*, and *SOC1*

**FIGURE 4:**
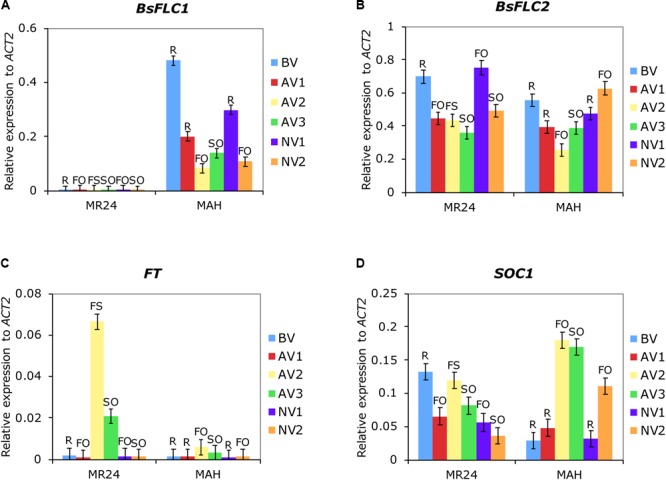
Expression patterns of four flowering-related genes **(A)** *BsFLC1*, **(B)** *BsFLC2*, **(C)** *FT*, and **(D)** *SOC1*. Shown are the means and standard errors of relative expression to reference gene *ACT2* from two parental accessions and six time points. For each graph, the horizontal axis first separates the two accessions (MR24/MAH) then the six time points (BV to NV2: BV – before vernalization, 7-week plants. NV1 – no vernalization group 1, 9-week plants. NV2 – no vernalization group 2, 18-week plants; AV1 – after vernalization group 1, 1 week after vernalization; AV2 – after vernalization group 2, 3.5 weeks after vernalization; AV3 – after vernalization group 3, 6 weeks after vernalization. Detailed timeframe in Supplementary Figure S1). Capital letters above each bar represent the stage of experimental plants (R, rosette; FO, flowers only; FS, with flowers and siliques; SO, siliques only).

While the previous F_2_ linkage analysis showed the genomic region containing *BsFLC1* deletion is strongly associated with rapid phenology, many genes exist in this genomic region. To find further support that *BsFLC1* is associated with flowering time but *BsFLC2* is not, we used qPCR to characterize the expression pattern of the two genes, as well as *FT* and *SOC1*, two floral pathway integrators that are directly inhibited by *FLC* in *Arabidopsis* (Helliwell et al., 2006). Replicated individuals from two parental accessions (MR24 and MAH) were sampled across six time points with or without vernalization (Supplementary Figure S1). Due to the deletion, *BsFLC1* in MR24 had no qPCR signal (**Figure 4A**). Both genes have similar levels of expression during the rosette stage before vernalization. The expression of *BsFLC1* in MAH associated strongly with plant stage: high expression in vegetative stage but low in reproductive stage (**Figure 4A**). In rosettes, *BsFLC1* expression was suppressed by vernalization (**Figure 4A** from time point BV to AV1) and decreased through time even without vernalization treatment (**Figure 4A** from time point BV to NV1 to NV2). On the other hand, the expression pattern of *BsFLC2* had no clear association with plant stages. In fact, the high expression of *BsFLC2* in some reproductive stages suggested this gene did not retain the ancestral function to suppress flowering (**Figure 4B**). *BsFLC2* was also suppressed by vernalization in either accessions with or without *BsFLC1*, although the magnitude of suppression is not as much as *BsFLC1* (**Figure 4B** from time point BV to AV1).

In the MAH accession with functional *BsFLC1*, the expression pattern of two downstream floral integrators, *FT* and *SOC1*, also associated strongly with plant stages (**Figures 4C,D**). Opposite to *BsFLC1*, both genes had higher expression during reproductive but lower expression in vegetative stages, although the pattern in *FT* was less clear due to its generally lower expression. In addition, the correlation between *BsFLC1* and *SOC1* expression was strongly negative across these six time points (Spearman’s ρ = -0.94, *P* = 0.005, **Table 2**). In the MR24 accession with *BsFLC1* deletion, however, the association between *FT* or *SOC1* and plant stage was disrupted (**Figures 4C,D**). *BsFLC2* had no significant correlation with the two downstream genes in either MAH or MR24 (**Table 2**).

**Table 2:** Pairwise correlation (Spearman’s ρ, with *P*-values in parentheses) between the expression patterns of four genes from six time points in two accessions (upper triangle, MAH; lower triangle, MR24, the accession without *BsFLC1*).

*BsFLC1*	0.43 (0.397)	–0.43 (0.397)	–0.94 (0.005)
–	*BsFLC2*	–0.71 (0.111)	–0.66 (0.156)
–	–0.43 (0.397)	*FT*	0.60 (0.208)
–	–0.26 (0.623)	0.66 (0.156)	*SOC1*

In summary, *BsFLC1* in *B. stricta* may have retained the ancestral flowering-related function: its expression has high association with the vegetative/reproductive plant stages, is reduced after vernalization treatments, and is correlated with downstream flowering genes. In contrast, *BsFLC2* may have diverged in function, although its expression was still suppressed by vernalization.

### Evolution of the Two FLC Copies After Gene Duplication

**FIGURE 5:**
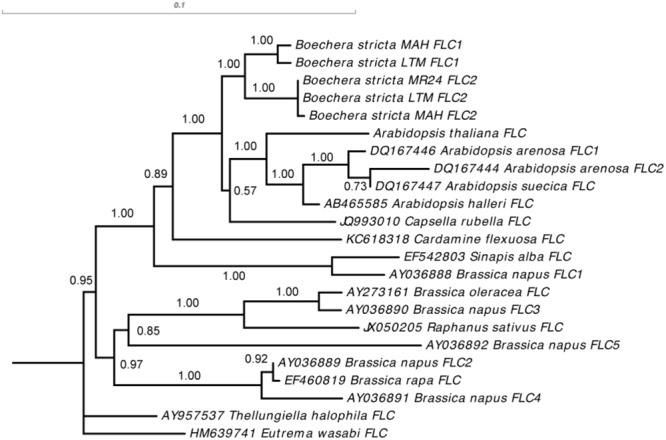
Phylogenetic reconstruction of *FLC* coding sequences in Brassicaceae, showing that the gene duplication event creating *BsFLC1* and *BsFLC2* in *Boechera stricta* happened after the divergence between *Boechera* and *Arabidopsis*. The number above each internal branch represents posterior probability from Bayesian inference method. For sequences obtained from GenBank, accession numbers are labeled in front of species names.

Phylogenetic reconstruction of *FLC* homologs across Brassicaceae showed clearly that the gene duplication event happened after the *Boechera*-*Arabidopsis* divergence (**Figure 5**). We therefore focused our analysis on *BsFLC1* and *BsFLC2* within *Boechera*, using *Arabidopsis FLC* as the outgroup. After duplication, *BsFLC1* accumulated three substitutions (one synonymous and two non-synonymous) while *BsFLC2* accumulated 10 substitutions (one synonymous, eight non-synonymous, and a three-codon deletion). Using all substitution types, Tajima’s relative rate test (Tajima, 1993) identified no strong evolutionary rate difference between the two copies (*x^2^*_1_ = 3.769, *P* = 0.052). Considering only non-synonymous substitutions, the evolutionary rate of *BsFLC2* is significantly higher than *BsFLC1* (*x^2^*_1_ = 4.455, *P* = 0.036). In addition, *BsFLC2* accumulated more non-synonymous substitutions than *BsFLC1*, resulting in much higher *d_N_*:*d_S_* ratio (0.608 for *BsFLC1* and 2.460 for *BsFLC2*, without considering the three-codon deletion in *BsFLC2*, **Table 3**). However, statistical tests using PAML showed that: (1) The *d_N_*:*d_S_* ratios do not differ significantly between *BsFLC1* and *BsFLC2* (*P* = 0.421); (2) The *d_N_*:*d_S_* ratio for *BsFLC2* is not significantly different from 1.0 (*P* = 0.235); and (3) There is little evidence that specific codons on the *BsFLC2* lineage were under positive selection (*P* = 0.236).

**Table 3:** Synonymous and non-synonymous substitutions in *BsFLC1* and *BsFLC* after gene duplication.

Gene	S differences	S sites	*d_S_*	N differences	N sites	*d_N_*	*d_N_*/*d_S_*
*BsFLC1*	1	134.33	0.0074	2	444.67	0.0045	0.6081
*BsFLC2*	1	134.50	0.0074	8	444.50	0.0182	2.4595

*This table compares the difference between each gene and the ancestral sequence before gene duplication. Columns: S differences, number of synonymous differences; S sites, number of synonymous sites; d_*S*_, S differences/S sites; N differences, number of nonsynonymous differences; N sites, number of nonsynonymous sites; d_*N*_, N differences/N sites.*

The distribution of amino acid substitutions after gene duplication is not random (Supplementary Figure S5). The two amino acid substitutions on *BsFLC1* do not have an obvious spatial concentration. In contrast, all *BsFLC2* changes are concentrated on or near the K-box domain. Assuming amino acid substitutions happened independently and randomly on *BsFLC2*, it is highly unlikely that eight out of nine amino acid changes would be located inside the K-box domain (*P* = 0.006), as only six out of one thousand resampling trials gave eight or more changes within the K-box domain. The types of amino acid substitutions also differed between the two *BsFLC* copies. While both *BsFLC1* substitutions and most substitutions on *BsFLC2* resulted in the change of amino acids with physicochemical distances less than 56 (Grantham, 1974), the *BsFLC2* lineage contained three nearby amino acid substitutions that greatly altered the physicochemical properties on these positions (distance of 99, 109, and 98 for amino acid positions 116, 118, and 121, respectively, Supplementary Figure S5).

## Discussion

### Repeated Evolution of Flowering Time Variation Through Change of FLC

While phenotypic changes can be achieved by segregation and recombination of standing genetic variants, mutations play an important role in generating novel phenotypes. Novel mutations differ in size (from Mendelian to polygenes) and direction (from advantageous, neutral, to deleterious) of their effects (Orr, 2005; Eyre-Walker and Keightley, 2007). Due to their unique roles in development or positions in biochemical pathways, genes with large effect may often be the targets of repeated evolution for similar phenotypes (Martin and Orgogozo, 2013). The *FLC* gene constitutes one example. In addition to *BsFLC1* in *B. stricta*, independent mutations in *FLC* homologs cause heritable variation in vernalization requirement of flowering time in other Brassicaceae species (Alonso-Blanco and Méndez-Vigo, 2014) such as *A. thaliana* (Michaels et al., 2003; Lempe et al., 2005; Shindo et al., 2005; Werner et al., 2005; Méndez-Vigo et al., 2011), *Arabidopsis lyrata* (Kemi et al., 2013), *Arabis alpina* (Wang et al., 2009; Albani et al., 2012), *Capsella rubella* (Guo et al., 2012; Yang et al., 2018), *Brassica napus* (Tadege et al., 2001), *Brassica oleracea* (Okazaki et al., 2007), and *Brassica rapa* (Schranz et al., 2002). Recently, *ODDSOC2*, the *FLC*/*MAF* ortholog in monocots, was also shown to be associated with the vernalization requirement of flowering in *Brachypodium distachyon* (Sharma et al., 2016).

Here we studied the genetic architecture of rapid phenology in *B. stricta* and investigated the two *FLC* homologs in *B. stricta*. We showed that the gene duplication event is specific to *Boechera* (**Figure 5**), and previous data have identified both *FLC* copies in multiple *Boechera* species (Schranz, unpublished data). *BsFLC1* is the homolog of *A. thaliana FLC* in the syntenic region. This genomic region controls large variation in flowering time (**Figure 3**), and accessions with the *BsFLC1* deletion (**Figure 2** and Supplementary Figure S3) flowered rapidly regardless of vernalization treatment (**Figure 1**). We recognize that our vernalization treatment changed both temperature and photoperiod within biologically realistic limits, and there may be other genetic mechanisms affecting such response. In addition, the expression pattern of *BsFLC1* is similar to other Brassicaceae plants and highly associated with plant vegetative/reproductive stages and the expression pattern of downstream flowering genes (**Figure 4** and **Table 2**). In the annual plant *A. thaliana, FLC* expression is stably repressed epigenetically after vernalization and “reset” only during embryogenesis (Sheldon et al., 2008; Choi et al., 2009; Berry and Dean, 2015). In perennial plants such as *Arabis alpina*, expression of *FLC* is also repressed during but restored after vernalization, allowing plants to return to the vegetative stage (Wang et al., 2009; Kiefer et al., 2017). The expression pattern of *BsFLC1* is similar to the latter, consistent with the perenniality of *B. stricta*. Our analyses suggest that after gene duplication, *BsFLC1* likely retained the ancestral function to suppress flowering, and the evolution of rapid phenology in *B. stricta* accession MR24 happened through the deletion of *BsFLC1*.

### Change of *BsFLC2* Function Enables Evolution of Rapid Phenology Through *BsFLC1* Deletion

The other gene, *BsFLC2*, is the unlinked and duplicated paralog. The mismatch between *BsFLC2*’s expression pattern and plant vegetative/reproductive stage, as well as its strong expression in the fast-flowering MR24 accession, suggest it does not retain the ancestral function to control flowering (**Figure 4**). As a result, one deletion event in *BsFLC1* is enough to generate the loss-of-vernalization-requirement phenotype in MR24.

As to how or when *BsFLC2* lost the flowering-related functions, there are several possibilities (Introduction). First, only part of the gene was duplicated, and *BsFLC2* is a truncated non-functional gene. Our results show that *BsFLC2* is likely to be functional given its intact and in-frame coding sequence, the conserved MADS-box domain (Supplementary Figure S5), the retention of several kbs of ancestral sequence upstream and downstream (Supplementary Figure S6), and the ability to be suppressed by vernalization.

Second, the duplication event may disrupt the upstream or downstream regulatory sequence, causing *BsFLC2* to be expressed in completely different tissues or times, thereby altering the biological function of *BsFLC1*. We observe that the expression patterns between the two copies are qualitatively similar (**Figure 4**): both copies had similar levels of expression in young rosette leaves (where and when the flowering-suppression function happened) and were suppressed by vernalization. The duplication event therefore does not seem to dramatically change the expression pattern of *BsFLC2*. We note, however, that there are subtle differences between the two copies: *BsFLC2* expression was not suppressed by vernalization as much as *BsFLC1*, and it was highly expressed during the flowering stage for plants without the vernalization treatment (**Figure 4**). Since the precise regulation of *A. thaliana FLC* requires non-coding regions within and flanking the gene (Michaels and Amasino, 1999; Swiezewski et al., 2009; Sun et al., 2013; Csorba et al., 2014), *BsFLC2* might not retain enough ancestral regulatory sequences (Supplementary Figure S6). Therefore, it is possible that the duplication event may cause *BsFLC2* to exhibit slightly different expression patterns than *BsFLC1*, although it remains unclear whether such difference was directly caused by or happened after the gene duplication event.

While the two previous possibilities concern the duplication event directly disrupting the coding sequence or regulatory region of *BsFLC2*, our results are also consistent with the third hypothesis: the coding and regulatory functions of *BsFLC2* remained largely unaffected by the gene duplication event, and *BsFLC2* diverged in protein function afterward. We observed protein sequence evolution in *BsFLC2* that cannot be completely explained by neutral evolution following gene duplication. Depending on the methods used, we sometimes obtained mixed results regarding whether the evolutionary rate differs significantly between *BsFLC1* and *BsFLC2*. It should be noted that these results might be in part due to the lack of statistical power, given the limited number of nucleotide substitutions after this very recent gene duplication event. In addition, these *d_N_*/*d_S_*-based methods ignored the three-codon deletion in *BsFLC2*, which removed three amino acids in a functionally important domain (below).

The *FLC* protein is a MIKC-type protein, consisting of the MADS (M-), intervening (I-), keratin-like (K-), and C-terminal (C-) domains (Theißen et al., 1996; Kaufmann et al., 2005). The major function of MADS-box is DNA-binding (Kaufmann et al., 2005), and in *FLC* this domain is associated with chromatin interactions of *FT* and *SOC1* (Helliwell et al., 2006), two downstream floral pathway integrator genes. The other domains, especially the K-box domain, are associated with dimerization or protein-protein interaction (Kaufmann et al., 2005) and may be important to form the functional protein complex, as multiple *FLC* proteins were identified in the same multimeric protein complex (Helliwell et al., 2006). All amino acid substitutions in *BsFLC2* are concentrated near the K-box (Supplementary Figure S5), suggesting that the *BsFLC2* protein may have undergone functional change after gene duplication, interacting with other proteins.

In this study, we investigate the interplay between loss-of-function evolution and gene duplication. We show that *BsFLC1* and *BsFLC2* differ slightly in expression patterns, which might be caused either directly by the gene duplication event or by later mutations. More importantly, after gene duplication *BsFLC2* accumulated amino acid substitutions in a speed higher than neutral evolution and in a region more concentrated than random expectation, suggesting directional selection driving its protein sequence evolution. We propose such changes alter protein functions, allowing a single deletion event encompassing *BsFLC1* to create a novel loss-of-vernalization-requirement phenotype in the MR24 accession despite the continual expression of *BsFLC2* in rosette leaves.

## Data Accessibility

DNA sequences: GenBank MH166767-MH166771. DNA sequence alignment, phenotypic values, and qPCR results: uploaded as Supplementary Materials.

## Author Contributions

C-RL, MS, and TM-O designed the study. C-RL and J-WH conducted experiments. C-RL analyzed data and wrote the manuscript with help from all authors.

## Conflict of Interest Statement

The authors declare that the research was conducted in the absence of any commercial or financial relationships that could be construed as a potential conflict of interest.
