# Supplementary material for: The Functional Change and Deletion of FLC Homologs Contribute to the Evolution of Rapid Flowering in Boechera stricta
Source: Front Plant Sci. 2018 Jul 31;9:1078. doi: 10.3389/fpls.2018.01078 (PMC6080596; doi:10.3389/fpls.2018.01078)
Supplement: Supplementary file 1 [file Table_1.DOCX]

**Table S1** All primer sequences used in this study

| Name | Sequence | Gene | Direction | Note |
| --- | --- | --- | --- | --- |
| FLC-A | CGGTCTCATCGAGAAAGCTC | *BsFLC1 & 2* | Forward |  |
| FLC-B | CGATATGGGAAACAACATGC | *BsFLC1 & 2* | Forward |  |
| FLC1-C | CATCTCAGCTTCTGCTCCCA | *BsFLC1* | Reverse | Inside exon for genomic and cDNA qPCR |
| FLC2-C | TCTCCATCTCAGCTTCAACA | *BsFLC2* | Reverse | Inside exon for genomic and cDNA qPCR |
| FLC1-D | GCATGTATCTAGCTTTTCCCAG | *BsFLC1* | Reverse |  |
| FLC1-E | TGTTCAGCAATCACAATCTAAC | *BsFLC1* | Reverse |  |
| FLC-F | TGTTGAAGCTTGTTGAGAACCT | *BsFLC1 & 2* | Forward | Inside exon for cDNA qPCR |
| FLC-G | TGGCCAGGATCTTCAGTCAG | *BsFLC1 & 2* | Forward |  |
| FLC-H | TAAGGATCAAATTAGGGCACAA | *BsFLC1 & 2* | Forward |  |
| FLC-I | CGCCATTATCAGCGGAATA | *BsFLC1 & 2* | Reverse |  |
| FLC1-J | GGGAAAAGCTAGATACATGCTTCA | *BsFLC1* | Forward | Inside intron for genomic qPCR |
| FLC2-J | CTTTCTCATGCTTCCAAACTTAAAA | *BsFLC2* | Forward | Inside intron for genomic qPCR |
| FLC-K | GACCGCCCTCTCCGTAACTA | *BsFLC1 & 2* | Forward | Inside exon for cDNA qPCR |
| BsACT2-CL-A | TGCTGTTGTGGTGAACATGTAA | *ACT2* | Reverse | Inside exon for genomic and cDNA qPCR |
| BsACT2-CL-B | TGATTCTCTTCCCTTGAACAGG | *ACT2* | Forward | Inside intron for genomic qPCR |
| BsACT2-CL-C | CGTACAACCGGTATTGTGCTG | *ACT2* | Forward | Spans intron for cDNA qPCR |
| ACTIN2LTM-FW | TCACCACAACAGCAGAACGGGAAA | *ACT2* | Forward | For RT-PCR |
| ACTIN2LTM-RW | TGCTGGAAAGTGCTGAGAGAAGCA | *ACT2* | Reverse | For RT-PCR |
| BsFT-CLqPCR-A | ATCCCTGCTACAACTGGAACAA | *FT* | Forward | Inside exon for cDNA qPCR |
| BsFT-CLqPCR-B | GCAGCCACTCTCCCTCTGAC | *FT* | Reverse | Inside exon for cDNA qPCR |
| BsSOC1-CL-A | TGCAACAAGCAGACAAGTGAC | *SOC1* | Forward | Inside exon for cDNA qPCR |
| BsSOC1-CL-B | TGGTATCTTGCATATTGGAGCTG | *SOC1* | Reverse | Spans intron for cDNA qPCR |
| JGI13175-36-F | TGGTTTTTGTCCATCCCAAC | microsatellite | Forward | Polymorphic between MR24 and MAH |
| JGI13175-36-R | CATGCCAATTGTGGAATCTG | microsatellite | Reverse | Polymorphic between MR24 and MAH |
| BsFLCdel-2 | TGCATGAATCACGTGAAGAGC |  | Forward | Confirm the extent of deletion |
| BsFLCdel-3 | CTCCGACGCCATGAGAATCA |  | Reverse | Confirm the extent of deletion |
| BsFLCdel-8 | CTGTCGCTTGGAAGGACGTA |  | Forward | Confirm the extent of deletion |
| BsFLCdel-9 | CCGAAATACTCGAAACCGGG |  | Reverse | Confirm the extent of deletion |
