# Supplementary material for: The Functional Change and Deletion of FLC Homologs Contribute to the Evolution of Rapid Flowering in Boechera stricta
Source: Front Plant Sci. 2018 Jul 31;9:1078. doi: 10.3389/fpls.2018.01078 (PMC6080596; doi:10.3389/fpls.2018.01078)
Supplement: Supplementary file 3 [file Image_1.PDF]

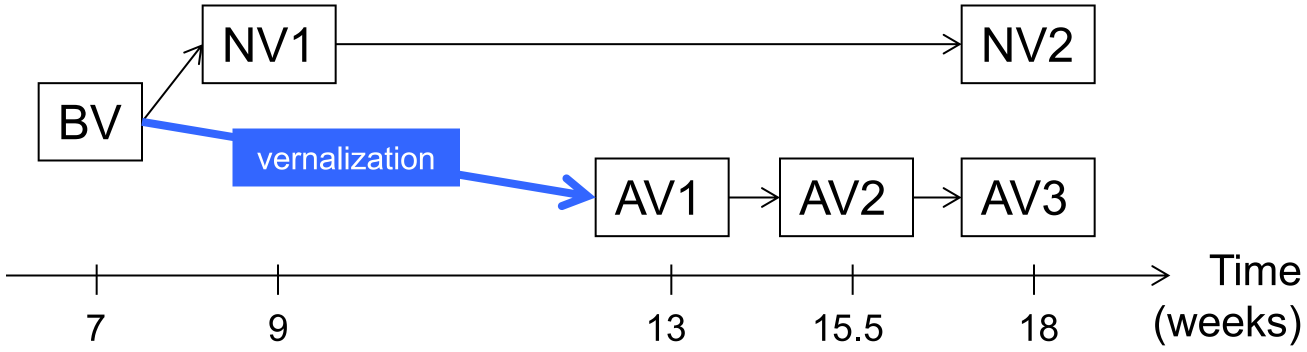

	BV	NV1	AV1	AV2	NV2	AV3
MR24	R	FO	FO	FS	SO	SO
MAH	R	R	R	FO	FO	SO

Figure S1. The six time points (labeled in boxes) where the expression patterns of *BsFLC1*, *BsFLC2*, *SOC1*, and *FT* were analyzed in the MR24 and MAH genotypes. Horizontal axis labels the weeks after seeds planted. The blue arrow denotes the six-week vernalization treatment in 4 ° C, 10-hour days. The table below lists the stage of the two plant genotypes in each time point. R – rosette. FO – flower only. FS – with flower and silique. SO – silique only.

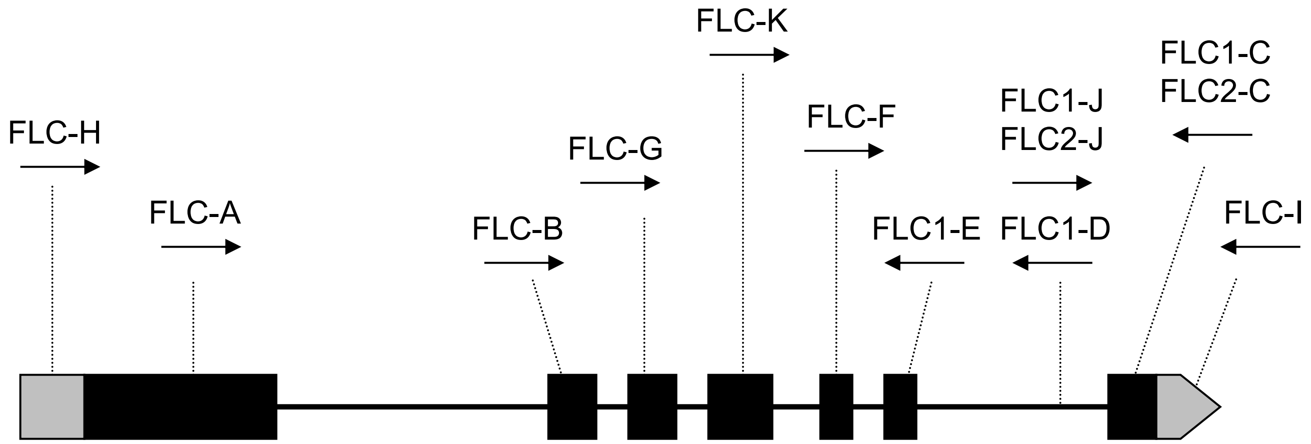

Figure S2. Primers and their location on *FLC*. Gene model was drawn based on *Arabidopsis*. Black boxes are coding regions, grey boxes are UTR (arrow represents the direction of transcription), and horizontal lines are introns (not drawn to scale). Arrow points to the direction of each primer.

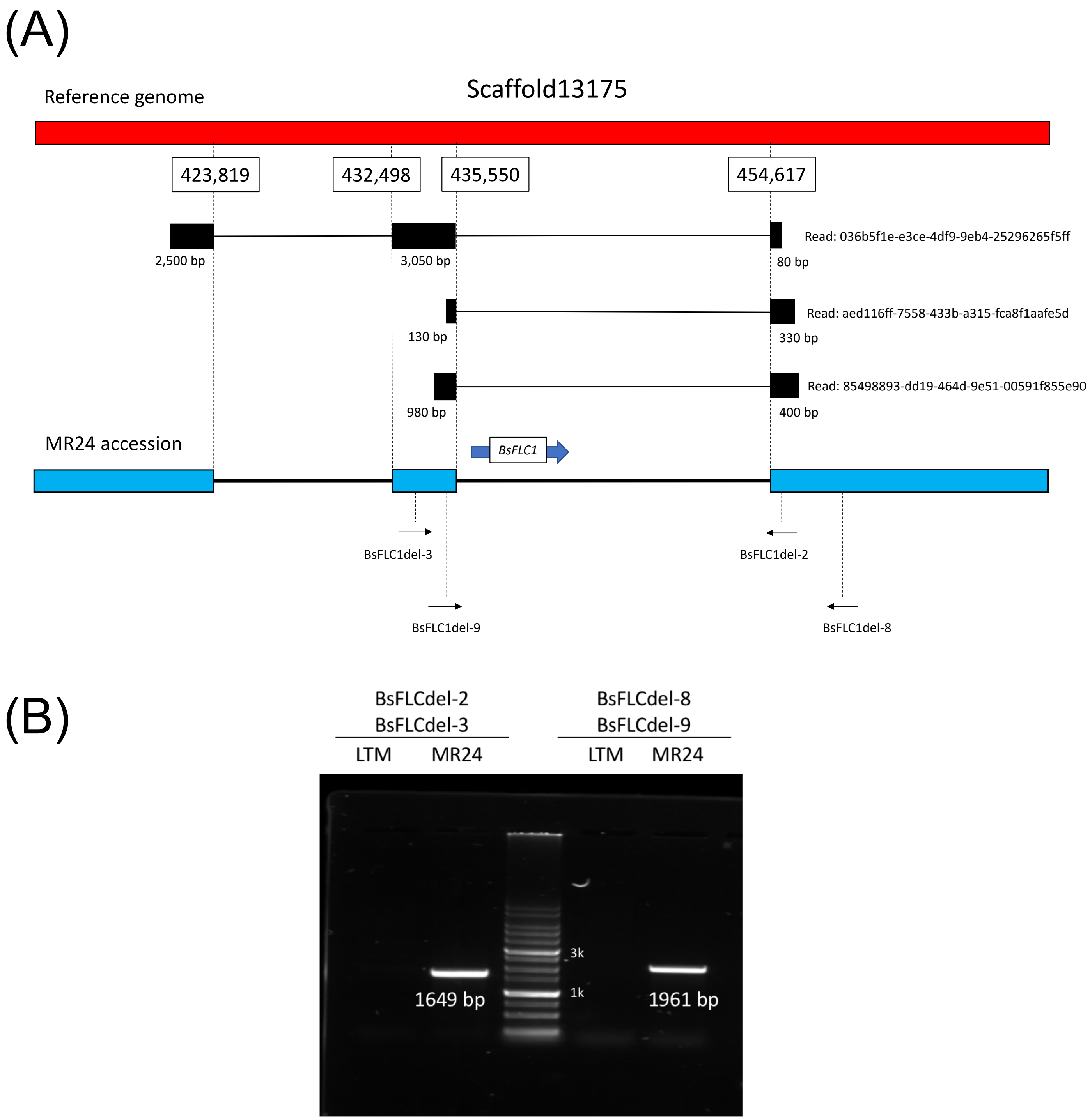

Figure S3. (A) Identification of the deletion by three Nanopore reads. The deletion in accession MR24 is about 19 kb long and covers *BsFLC1*. Another indel on the left was also identified, which is likely a LTM reference genome specific insertion, as it does not exist in the draft *B. retrofracta* (the sister species of *B. stricta*) scaffold. (B) Confirmation of the extent of deletion by two pairs of primers, whose locations are shown in panel (A).

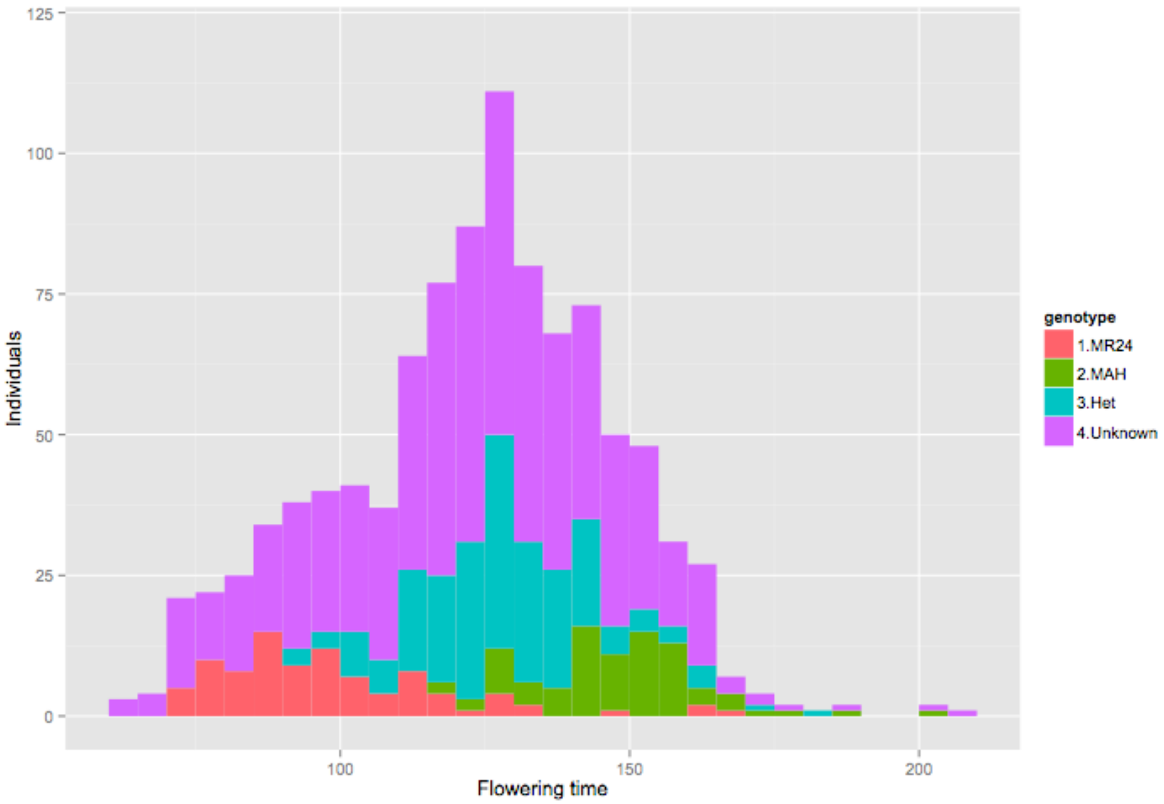

Figure S4. Flowering time distribution of 1,000 F2 individuals from the cross between MR24 and MAH genotypes. 384 individuals were genotyped for the microsatellite marker JGI13175-36, and the genotypes are labeled. For the two parents, in average MR24 flowered at 97 days, and MAH flowered at 155 days. Both parents have standard errors of 2 days. The microsatellite explains 70% of phenotypic variation.

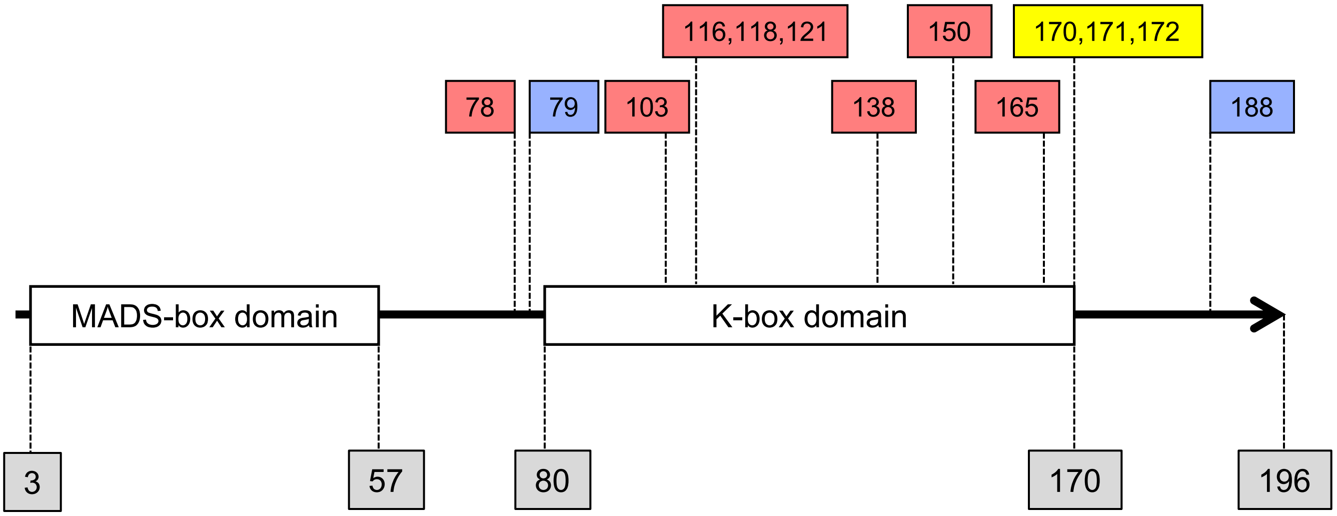

Figure S5. Amino acid substitutions in *BsFLC1* and *BsFLC2* after gene duplication. Shown are the amino acid positions in the *FLC* protein. White boxes are protein domains, grey boxes denote domain boundaries, and the yellow box denotes a 3-codon deletion in *BsFLC2*. Blue and red boxes are amino acid substitutions in *BsFLC1* and *BsFLC2* respectively.

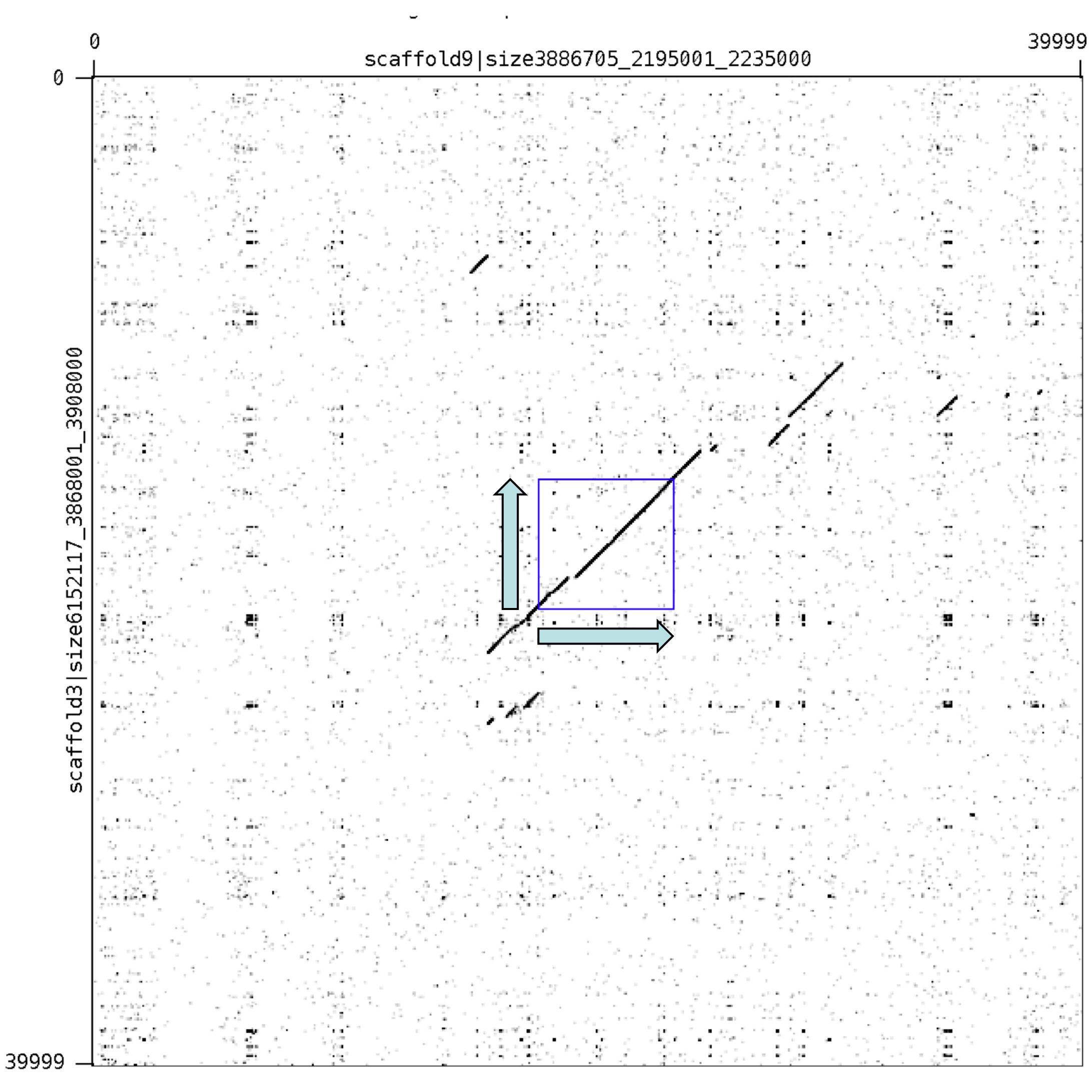

Figure S6. Dotplot alignment of two *Boechera retrofracta* scaffolds containing *BsFLC1* (on scaffold3) and *BsFLC2* (on scaffold6) orthologs in *B. retrofracta*. The blue square covers the *FLC* gene region, and arrows represent gene orientation. The ~2 kb region upstream of *FLC* is syntenic between the two copies.
